# Zebrafish: A Promising Real-Time Model System for Nanotechnology-Mediated Neurospecific Drug Delivery

**DOI:** 10.1186/s11671-021-03592-1

**Published:** 2021-08-23

**Authors:** Suraiya Saleem, Rajaretinam Rajesh Kannan

**Affiliations:** Neuroscience Lab, Centre for Molecular and Nanomedical Sciences, Centre for Nanoscience and Nanotechnology, School of Bio and Chemical Engineering, Sathyabama Institute of Science and Technology (Deemed to be University), Jeppiaar Nagar, Rajiv Gandhi Salai, Chennai, Tamil Nadu 600119 India

**Keywords:** Zebrafish, Drug delivery, Blood–brain barrier, Nanotechnology, Nanoparticles

## Abstract

Delivering drugs to the brain has always remained a challenge for the research community and physicians. The blood–brain barrier (BBB) acts as a major hurdle for delivering drugs to specific parts of the brain and the central nervous system. It is physiologically comprised of complex network of capillaries to protect the brain from any invasive agents or foreign particles. Therefore, there is an absolute need for understanding of the BBB for successful therapeutic interventions. Recent research indicates the strong emergence of zebrafish as a model for assessing the permeability of the BBB, which is highly conserved in its structure and function between the zebrafish and mammals. The zebrafish model system offers a plethora of advantages including easy maintenance, high fecundity and transparency of embryos and larvae. Therefore, it has the potential to be developed as a model for analysing and elucidating the permeability of BBB to novel permeation technologies with neurospecificity. Nanotechnology has now become a focus area within the industrial and research community for delivering drugs to the brain. Nanoparticles are being developed with increased efficiency and accuracy for overcoming the BBB and delivering neurospecific drugs to the brain. The zebrafish stands as an excellent model system to assess nanoparticle biocompatibility and toxicity. Hence, the zebrafish model is indispensable for the discovery or development of novel technologies for neurospecific drug delivery and potential therapies for brain diseases.

## Introduction

Drug delivery refers to the method of transferring compounds into the body for therapeutic purpose. The compounds are mainly pharmaceutical in nature and targeted against a particular disease condition to a particular cell population in vivo. The term drug delivery encompasses two main ideas: form of dosage and route of administration [1]. Proper drug delivery ensures efficient drug activity by regulating the following: drug release, absorption by cells and correct distribution within the system [2]. Some common drug delivery routes include enteral (gastrointestinal tract), parenteral (via injections), inhalation (olfactory mediated), transdermal (via dermis), topical (through skin) and oral routes (via oesophagus) [3]. Delivering a drug is critical and of major significance in the field of therapeutics. The chosen method must be most effective and also least toxic to the system [4]. The problem becomes even bigger when the organ in question is the brain. Delivering drugs to the brain has been a struggle among researchers for over decades now [5, 6]. Innumerable technologies and ideas have been used for the development of an effective technique [7, 8]. Yet, success doesn’t seem too near. The biggest hurdle in this struggle is the ability to cross the blood–brain barrier (BBB). The BBB is a physiological barrier to protect our brains from compounds being transferred from blood to the brain [9]. The natural makeup of the barrier allows only very small molecules in the blood stream to have access into the brain [10]. Molecules with small molecular weights < 400 Da and those which are lipid soluble have the ability to penetrate the brain [11]. Neurospecific drugs must meet these parameters for effective drug delivery across the BBB. At present, most of the drugs developed to target the brain are unsuccessful in crossing the BBB [9, 12, 13]. Diseases of the central nervous system are some of the most prevalent diseases affecting several people in all stages of life. However, these diseases still remain the least treated [14]. There is an immediate need for novel neurospecific drug delivery technologies since the success rates of existing drugs targeted to the brain is extremely low. Apart from the restricted permeability of the BBB, the complexity of the brain and the side effects caused by existing drug delivery technologies need to be taken care of as well [15]. Absence of an absolute method for efficient delivery of neurospecific drugs has hindered effective drug development in this field. The research community has explored various avenues for delivering safe and targeted drugs to the brain. Macromolecules to nanoparticles are being explored to ensure maximum effectiveness [16].

Nanotechnology has increasingly acquired the interest of the scientific community by its booming impact on research on brain drug delivery [17]. With the growth in nanotechnology, there has been a simultaneous expansion of the nanotoxicology sector. The toxicity assessment of the nanoparticles plays a pivotal role in analysing the impact of the nanoparticles to the individual species and the environment at large [18]. Recent years have seen the application of zebrafish as a prototype for toxicity studies [19]. The zebrafish has been used extensively for studies on experimental biology and is now evolving as a robust model system to study nanotoxicity [20]. In terms of model system for nanotoxicity, the zebrafish offers several advantages. It is highly economical to use as an experimental animal and easy to maintain. It has high fecundity rate making them easily available and helping to understand the vertebrate physiology in an easier way [21]. However, the use of zebrafish as a model system has its limitations too. First and foremost, the nervous system of the zebrafish may not be as complicated and developed as that in humans; the rodent and murine nervous systems are comparatively better developed and can be used to study the complex human brain diseases; however, they are not identical to that of humans [22]. Secondly, the zebrafish lacks some organ systems found in humans like the lungs, prostate and the mammary glands; also, diseases caused by genes absent in the zebrafish cannot be studied [23]. However, the zebrafish shares 70% genomic similarity with the human genome and 84% homology with human disease causing genes which makes it highly suitable to mimic human disease pathology [24]. The adult zebrafish had previously been postulated to be devoid of the liver macrophage; the Kupffer cells were regarded to be present only transiently in the early embryonic stage and absent or sparse in later stages of development [25–27]. However, recent work has shown the hematopoietic origin of the Kupffer cells and their persistence even in the adult zebrafish liver making zebrafish adept for research on Kupffer cells as well [28, 29]. Further, the higher vertebrate models are expected to mimic the complicated human pathologies to greater accuracy than the zebrafish. Recently, a debate has commenced over the reliance on data available from animal models and their extrapolation to humans [30]. This points out to the fact that any animal model whatsoever has its own limitations when applied to clinical studies [30, 31].

This review discusses the most recent studies on nanotechnology-mediated drug delivery specifically to the brain using the zebrafish as a model system. It summarizes the hurdles of the BBB and the various nanodrug optimizations, their toxicity evaluation and impact as use for therapeutics in neurodegenerative diseases using both zebrafish embryos and adults. Finally, the review highlights the advantages and disadvantages of the zebrafish model for neurospecific drug delivery and brings to light the immense scope it holds for future translational research.

## Blood–Brain Barrier: The Main Obstacle in Neurospecific Drug Delivery

The BBB ensures restricted entry of substances into the brain, hence acting as a diffusion barrier helping to maintain normal brain homeostasis [32]. Several cells are involved in making up the composite structure of the BBB [33]. Pericytes, astrocytes and neurons comprise the cellular components, while the endothelial cells, tight junctions and basal membrane together constitute the BBB [34]. Lack of fenestrations in the endothelial cells in the brain ensures no diffusion of small molecules across their surface. Even water-soluble substances are hindered from entering the brain by the presence of inter endothelial junctions like tight junctions, adherens junctions and gap junctions, linking the endothelial cells [35]. These endothelial cells are in turn surrounded by the pericytes, astrocytes and basal membrane which complete the structure of the BBB [36]. The adherens junctions and tight junctions regulate the permeability of the endothelial cell layer. Gap junctions comprise of connexin molecules, and they control the communication between endothelial cells [37]. Molecules can cross the BBB via two pathways: the paracellular pathway or the transcellular pathway [38]. In the paracellular pathway, the ions and molecules pass the BBB by diffusing passively in between the cells using a concentration gradient [39]. The transcellular pathway employs the use of various mechanisms like transcytosis or receptor-mediated transport for passage of molecules through the cells [40]. Several parameters influence the permeability of the BBB. Molecular weight, charge on the surface, surface activity, solubility of the molecule and relative size of the molecule impact the BBB permeability [41].

### Blood–Brain Barrier: Modern Technologies for Drug Delivery

The blood–brain barrier (BBB) in a healthy brain mainly operates as a diffusion barrier to protect normal brain functions. It prevents most of the compounds from being transferred from the blood to the brain. The stringent BBB allows only very small molecules to enter the brain; however, it is observed to be disrupted in disease conditions.

### Why Nanoparticles Are a Current Choice for Neurospecific Drug Delivery

**Fig. 1:**
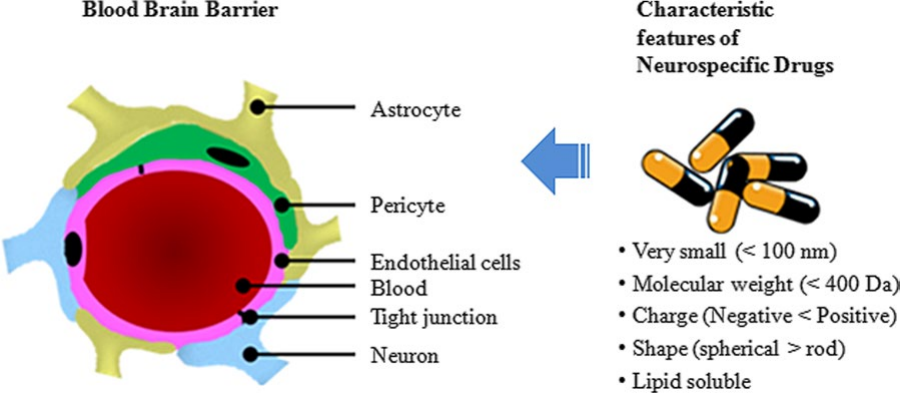
Characteristics of neurospecific drugs. The BBB is typically made up of the tight junctions in the endothelial cells surrounded by the astrocytes, pericytes and neurons. Neurospecific molecules should possess specific characteristics to be able to cross the blood–brain barrier (BBB). Preferred characteristics are: very small size with a diameter of less than 100 nm, low molecular weight preferably less than 400 Da, should be positively charged, spherical in shape and lipid solubility

The technique of engineering and synthesizing materials at the molecular level is referred to as nanotechnology. The National Nanotechnology Institute defines nanotechnology as any material which exists in at least one dimension and ranges in size between 1 and 100 nm (Fig. 1). The last decade has seen a boom in the field of nanotechnology and its applications in the biomedical field. Nanotechnology-based drug delivery is believed to have stirred up the entire biotechnology and pharmaceutical industries and bring a profound change in this field in the coming years [42–47]. The application of nanotechnology promises several advantages in targeted drug delivery. These include the ability to deliver drugs (a) of less water solubility to their respective target site, (b) of two or more types for achieving combinatorial therapy, (c) targeted delivery at the specific site of action, (d) transport of drugs across tight barriers, i.e. blood–brain barrier, (e) visualization opportunities for better understanding and analysis of drug activity [48] and (f) real-time tracking facility for achieving perfect efficacy in mode of drug activity [44]. Thus, nanotechnology technique holds tremendous potential for neurospecific therapeutics.

## Zebrafish as a Model for Neurospecific Drug Delivery

The *Danio rerio* (zebrafish) is a demonstrated vertebrate model for exploring development studies and the study of degenerative diseases [49–52]. It can be modelled for far ranging analyses, from fundamental and toxicological analysis to pre-clinical studies [53–55]. Of the several advantages offered by the zebrafish, its cost-effective maintenance, easy testing with simple housing requirements and a large clutch size are highly suited for high throughput testing [56]. High fecundity is a distinctive feature which further accentuates use of this model system [24, 57]. The organ systems of zebrafish are highly conserved to that of higher vertebrates [58].

The zebrafish embryos have external development and are completely transparent as a result of which they can be extensively visually studied. Thus, they are an excellent tool for screening analyses using agents that disrupt normal growth, development and cell cycle [59]. They display thorough development patterns ranging from epiboly to final development of key structures [60, 61]. Zebrafish are now used extensively for neuropsychiatric research and various studies to analyse developmental toxicity in nanoparticle-mediated drug delivery. Exposure of zebrafish to gold nanoparticles disrupted normal eye development and pigmentation as observed via a simple light microscope [62, 63]. Administering gold nanoparticles to zebrafish resulted in genotoxic effects and serious alterations in their genome constitution [64]. The dose- and time-dependent toxicity of silica NPs was determined by analysing its impact on the cardiovascular system [65, 66] and on the mortality rates [67]. It was also found that chitosan NPs have higher compatibility as compared to normal chitosan [68].

It is absolutely vital that nanoparticles used for clinical interventions must be biodegradable and non-toxic. Nanoparticles have great potential in the field of targeted drug delivery and translational research. The use of nanoparticles has been applied to an increasingly large number of fields including in vivo applications. This wide increase in the use of the nanoparticles implicates the lurking danger of excessive exposure of these nanocarriers to humans. Toxicity studies of the nanoparticles are an indispensable part of nanotechnology. Studies focusing on nanoparticle interactions at the cellular and molecular levels must be undertaken to analyse the toxicity before they can be clinically used. Table 1 summarizes the neurotoxicity studies of diverse nanocarriers employed for brain targeted drug delivery using zebrafish. Nanoparticle toxicity involves analysing the toxicity, permeability, rate of mortality, induced teratogenicity, immune reactions and genomic toxicity.

**Table 1:** A list of research done on nanocarrier neurotoxicity studies using zebrafish

Sr. no	Nanoparticle	Study highlights	References
1	Metal and metal oxide NPs		
	Au	Relates the effects of gold nanoparticles on zebrafish development, movement and survival	[64, 69–74]
	Ag	Provides detailed information on the toxicity and usage of silver nanoparticles	[75]
	Cu	The study highlights the fact that Cu nanoparticles are extremely toxic to the zebrafish. The toxicity primarily affects the gills	[76]
	Cd	Ventures into the use of cadmium nanoparticles into various commercial applications and assessing its toxicity using zebrafish	[77]
	CuO	Provides extensive information of copper oxide nanoparticles on cytotoxic impact on zebrafish	[78]
	MgO	Highlights the toxic effects of magnesium oxide nanoparticles on zebrafish	[79]
	NiO	Reports on the potential chronic toxicity caused by nickel oxide nanoparticles and the negative impact on the aquatic population dynamics	[80]
	ZnO	Brings to light the high toxicity of zinc oxide nanoparticles on zebrafish development. Presses on the need for eco-toxic evaluation of these nanoparticles	[81, 82]
2	Magnetic NPs	Fe_3_O_4_ magnetic nanoparticle exposure in adult zebrafish caused perturbations in neurotransmitter levels	[83]
		Dextran-coated iron oxide nanoparticles in brain of adult zebrafish alter acetylcholine esterase activity	[84]
		In this study toxicity of Iron oxide NPs on the aquatic environment has been studied extensively	[85]
		Study highlights the toxicity of iron oxide nanoparticle on the developmental stages of zebrafish	[85]
3	Graphene oxide nanosheets	Unfolds new technique for evaluating toxicity of nanomaterials by the use of fluorescence	[86]
4	Microplastics (MPs) and nanoplastics (NPs)	Reports on the extensive bioaccumulation and toxicity caused by plastic nanomaterials on the aquatic life	[87]
5	Plastic NPs	The study suggests plastic NPs cause abnormal locomotor ability in the zebrafish	[88]
6	Polymeric NPs and nanocapsules	Passage of the polymeric and PEGylated-PLA NP across the BBB and bioavailability in the brain was assessed in a zebrafish model	[89]
		Transport of PEGylated-PLA nanoparticles across the blood–brain barrier model, entry into neuronal cells and in vivo brain bioavailability	[90]
		Conjugated polymer NPs have been used for neuroimaging and assessing dopamine levels in the brains of zebrafish larvae	[91]
7	Exosomes	Exosomes derived from brain endothelial cells can be used to carry drug into the brain	[92]
		Exosomes were used to deliver siRNA in the zebrafish brain to treat brain cancer	[93]
8	Liposomes	This study highlights liposome-mediated drug delivery to regulate macrophage function in the zebrafish larvae	[94]
		Injecting drug loaded liposomes in the zebrafish larvae to deliver drug to the macrophage cells	[95]
		Injecting clodronate via liposomes to obtain macrophage clearance in the zebrafish model	[96]
		The study performed in zebrafish model allows prediction of nanoparticle cell interactions and persistence time in mice models	[97]
		This study highlights the use of zebrafish as screening model for liposome-mediated clearance of macrophage cells	[98]
9	Metal organic frameworks (MOFs)	The study reports the effect of zirconium-porphyrin metal–organic framework on zebrafish neurodevelopment	[99]
		Acute toxicity of Copper MOFs were analysed using zebrafish model	[100]
		This is an extensive comparative study of toxicity of sixteen uncoated MOFs in the zebrafish	[101]
10	Carbon Nanotubes (CNTs)	Zebrafish exposed to single-walled CNTs were assessed for neurotoxicity in terms of change in levels of neurotransmitter, antioxidants, gene expression and biochemical responses	[102, 103]
		Developmental toxicity and biological response of multiwalled CNTs were studied in zebrafish embryos and larvae	[104]
		Biospectroscopy techniques were employed to study the effects of real-world CNPs exposed to zebrafish brain and gonads	[105]
		Perturbations in the metabolomic profile of zebrafish exposed to CNTs were studied	[106]
		Carbon NPs from diet have been found to cause genomic hypermethylation of the zebrafish brain	[107]
11	Quantum dots	Graphene oxide quantum dots inhibit neurotoxicity and oxidative stress in zebrafish larvae	[108]
		The potentials of transferrin conjugated carbon dots in crossing the BBB were analysed using the zebrafish model	[109]
		Quantum dots have been used as labelling agents in the zebrafish embryos	[110]

Zebrafish is extensively used as a model system to evaluate nanoparticle toxicity and biocompatibility [111–113], and it holds great potential as a model for studying neurotoxicity and high throughput screening of nanoparticles [114–117]. No model other than the zebrafish is so aptly suited for such analyses. This model system can be used to study, analyse and manage the risks arising from toxicity of nanomaterials. The information gained will be helpful in formulating specific guidelines, framing protective measures and quality controls while working with nanotechnology-related products [118, 119].

### Insights on Nanoparticles-Mediated Drug Delivery Using Zebrafish Embryos

In order to use nanoparticles for targeting the brain, a prior knowledge on their effects in vivo is essential. The zebrafish model is best suited for this purpose. Recent studies have been conducted using nanoparticles to gain vital insights into hatching of zebrafish larvae. Use of TiO_2_ nanoparticles induces early hatching in the larvae in a dose-dependent manner [120]. Chen et al. suggest that TiO_2_ nanoparticles have an impact on larval swimming behaviour affecting both velocity and the level of activity [121]. On the other hand, Ong et al. reported complete inhibition of hatching and embryonic death of larvae upon exposure to nanoparticles. They further added that the cause of death of embryos is the physical interaction of the nanoparticles with the embryos rather than the effects of the physico-chemical properties of the nanoparticles [122]. Disruption of the thyroid endocrine system in the zebrafish larvae has also been observed when they are exposed to TiO_2_ nanoparticles [123]. Accumulation of lead has been attributed to be the cause of this adverse effect. TiO_2_ nanoparticles have also been reported to significantly activate levels of expression of BDNF, C-fos and C-jun. Conversely, it was also found to have an inhibitory effect on genes such as p38, NGF and CRE resulting in the brain damage of zebrafish [124]. TiO_2_ nanoparticles have also been shown to have adverse effects on the reproductive potential of the fish causing 9.5% reduction in the number of the eggs released [125]. Vogt et al. further reported the chemical toxicity of the small molecule BCI when added to zebrafish embryos 24–48 h post-fertilization [126]. Ali and Legler et al. showed nonylphenol nanoparticle-induced malformations in the embryos even at low dose [127]. Usenko et al. evaluated carbon fullerene [C_60_, C_70_, and C_60_(OH)_24_]-induced toxicity using zebrafish embryos [128], while Daroczi et al. enumerated the protective potential of the same nanomaterial from ionizing radiation [129]. Neuroprotective effect of C_60_ fullerene derivative, dendrofullerene nanoparticle (DF-1), in the zebrafish embryos has also been reported by assessing its toxicity [129]. Administration of silica nanoparticles to the fish embryos resulted in enhanced mortality [67], while ZnO nanoparticles increased mortality and also caused skin ulceration with delay in hatching [82]. The impact of exposure of waterborne nanoparticles on genes which regulate the immune system was first reported by Brun et al. [130]. This study highlights the importance of molecular responses as indicators of biological toxicity. Zebrafish embryos engrafted with cancer cells and subjected to polymersome nanoparticle have been imaged real time to understand nanoparticle toxicity and treatment strategy [131].

Interestingly, bio-imaging using zebrafish embryos of various developmental stages revealed the toxic effects of sodium cholate enclustered Ag nanoparticles [132, 133]. This study holds immense importance [134] as it shows that toxicity arising from Ag nanoparticles affects the gills and lamelli development in the fish. This inhibitory effect is mainly caused by interaction of Ag ions in the gills where they block the Na + /K + ATPase activity [135, 136]. Further, it is reported that Cu nanoparticles have a similar inhibitory effect on the growth of gills in the fish [76]. Use of copper nanoparticles in the larvae led to malformation and delayed hatching [69, 76]. Application of gold nanoparticles had no toxic effect on the larvae [69], while silver nanoparticle affected development [137]. Nanoparticles made of zinc, magnesium, iron, copper and nickel had no toxicity on the adults, but in the larvae, delayed hatching has been observed [78, 79, 81, 82, 138]. Nanoparticles of organic compound fullerene have also been shown to be nontoxic to larvae at concentrations below 200 mg/L [139]. Furthermore, it was also shown that nanoparticles of chitosan were far more effective and non-toxic as compared to the usual chitosan particles [68].

Metal oxide nanoparticles like TiO_2_ have been reported to induce some developmental malformations in the zebrafish larvae [120], while some report that it is completely non-toxic [140, 141]. The crucial parameter here is the dosage as well as time of exposure. Higher doses of the TiO_2_ NPs prove to be fatal for the larvae with accumulations of the NP in the gill, heart, liver and brain [141, 142]. Genotoxic effects are also a result of exposure to high doses of TiO_2_ to the fish [143]. Chronic exposure to lower concentrations (< 4 mg/L) of TiO_2_ NPs leads to lower toxicity and a higher mortality rate [142]. Another important feature of a nanoparticle to be taken into concern is the shape of the nanoparticle and the proteins on its surface. Grain-column hexagonal crystals of ZnO NPs impacted the zebrafish cell cycle [144], whereas ZnO NPs which were leaf-shaped and coated with polymer displayed higher biocompatibility as compared to the spherical NPs [122]. Furthermore, it has been shown that nanosticks are more toxic than spheres and cuboidal nanoparticles [145]. Iron NPs lead to severe deformities in the larvae [146] and genotoxic effects in the adults [134], whereas metals like nickel, cobalt and aluminium NPs are proved to be relatively inert [82, 147].

Keeping in view the increased devastation caused by plastic in today’s world, Pitt et al. showed its impact on the zebrafish. They observed that the developing zebrafish are highly susceptible to the nanoplastic available in the aquatic ecosystems. These nanoparticles can penetrate the chorion and have dismal impact on their physiology and behavioural responses [148]. This study goes on to elucidate the nuisance created by plastic to the underwater world which in turn impacts human civilisation. Research suggests that very small nanoparticles which have high surface area/volume ratios are highly capable of absorbing pollutants from the environment. The use of polystyrene nanoplastic beads in cosmetic products has been studied for their developmental toxicity and impact on zebrafish embryos [149]. Another study on polystyrene nanoplastic of less than 20 nm size has shown that it accumulates in the brain of the embryos [150].

### Insights Revealed by Nanoparticle Studies on Adult Zebrafish

A relatively extensive repertoire of research has been performed on the effects of nanoparticles on adult zebrafish. It acts as a valuable source of information on the use of nanoparticles in vertebrates. Truong et al. evaluated behavioural abnormality arising in 122 dpf embryos from exposure to gold nanoparticles [151]. Drug delivery to skin has also been accomplished by administering nanoparticles to the zebrafish. Researchers have shown that the Ag-BSA nanoparticles enter the skin by endocytosis where they accumulate and cause skin abnormalities [63]. Delivering drugs via nanoparticles have also been used to induce stress conditions in the zebrafish to act as potential models for drug discovery [152]. Some nanoparticles have been shown to induce asthma, apoptosis and enhanced immune response in the fish making it possible to use them for immunotoxicological studies [153–156]. Zebrafish model has been extensively studied for drug induced cardiotoxicity [157, 158]. The heart of a zebrafish exhibits several similar functional characteristics as that of a human heart including the pharmacologic drug responses [159–162]. The zebrafish heart is the first to develop at 22 hpf, while the entire cardiovascular system is ready by 48 hpf [163]. The zebrafish embryos have been visualized to study drug effects on heart rate, rhythmicity, contractility and circulation. Several visual assays have been performed using the zebrafish to help elaborate on cardiac health. A QT interval is one of the parameters on which most of the cardiac drugs are based on. The QT interval is the time gap between a Q and a T wave in the heart’s electrical cycle. A number of drugs have been assessed for their effect on QT interval (duration of ventricular action potential) using zebrafish [164–166]. One of the studies reported that drug causing prolongation of QT interval in humans actually leads to bradycardia and blocks auricular ventricular conduction [160]. The zebrafish liver forms by 48 hpf and becomes fully functional by 72 hpf; this model system is widely used to study liver based drug delivery. Studies in this field have revealed that the response exhibited by the zebrafish in hepatic toxicity is similar to that exhibited by the higher vertebrates [167]. The zebrafish have been used to characterize the orthologs of cytochrome P450, CYP3A and CYP3A65 [168, 169]. Further assessments have been performed to elaborate on the effect of drug on CYP3A4, CYP2D6 and CYP3A65 [170]. Neuroprotective effects of hesperetin nanoformulations have been studied in a traumatic brain injury model of zebrafish [171].

### Zebrafish Offers a Complete Pathological Study Model for Neurospecific Drug Delivery

When delivering drug to the brain, several adverse effects can take place. The zebrafish model offers the advantage of studying these in detail and hence provides a suitable technique of drug delivery to the brain [172]. Teratogenicity: Any kind of abnormal teratogenic growth or development can be easily assessed by observing the transparent zebrafish embryos [59]. Of the key perturbations that can be observed during teratoma formation are the pigmentation of the eye [67], mortality rates [65], changes in the cardiovascular system [68] and effects on hatching [115]. Immunotoxicity: Research has been conducted on the immunological reactions that arise in the zebrafish in response to drugs or nanoparticles. This leads to accumulation of neutrophils and macrophages [173]. The use of gold nanoparticles has been reported to disrupt inflammatory immune responses [174], while on the other hand silver nanoparticles have been shown to induce inflammatory responses [175]. Genotoxicity: Changes occurring at the DNA level can be observed by real-time PCR [143] and other comet assays [134]. Recent research on carbon-based NPs have attracted increased attention recently [176] mainly because of their low toxicity [177]. Carbon NPs are used in various forms in zebrafish which include fullerenes [128], carbon nanoparticles, carbon nanotubes (CNT) [178], graphene QDs [179] and carbon QDs (C-dots) [180]. Allotropes of carbon such as fullerenes have also been used as NPs since their discovery in 1985. They have been used extensively for drug delivery applications [181, 182]. Studies in zebrafish revealed that toxicity of fullerene NPs is dependent on the charge on its surface. Positively charged fullerenes were more toxic as compared to the negatively charged fullerenes [128]. Research shows that water soluble fullerenes have the capacity to protect against cell death by acting as free radical scavengers [129, 183]. Recent research has been done in zebrafish with nano-onions which are multi-shell fullerene structures. They exhibit low toxicity and good bio compatibility in the zebrafish larvae [184]. Carbon nanotubes (CNTs) possess distinct physico-chemical characteristics for which they are an attractive mode of drug delivery for researchers [176, 185, 186]. Efficiency of CNTs depends on their length and the nature of their walls, whether single or multi-walled. Reports suggest that single- or multi-walled pristine CNTs have minimal impact on the growth and development of the zebrafish larvae [187]. Variations in the length of the CNTs may lead to changes at the molecular level with longer CNTs being more cytotoxic [188]. Adult zebrafish when exposed to multi-walled CNTs have shown to exhibit toxicity including inflammatory gills [189] and accumulation of the CNTs in the brain and gonads [105, 190]. Another form of carbon-based NPs are the quantum dots (QDs) and graphene quantum dots (GQDs). The typical feature of the QDs is quasi-spherical carbon structures with a diameter of less than 10 nm [191] and that of GQDs is less than 30 nm [192, 193]. An additional feature of the QDs includes their unique photostability which enables it to combine with the fluorophores thus opening up a score of bioimaging possibilities [194]. QDs exhibit least toxicity as they are predominantly composed of inert carbon molecules [195]. Hence, a combination of fluoroluminiscence and low toxicity properties makes it a very attractive tool for drug delivery [195–197].

## Nanoparticles Focused for Delivering Drug to the Brain

With the background knowledge on action of nanoparticles on the physiology of zebrafish, researchers are now trying to deliver drugs to the brain via nanotechnology using the zebrafish models Table 2. Qian et al. have reported polymer nanoparticles conjugated with tags of phenylboronic acid on their surface which helps detect fluorescence for the neurotransmitter dopamine using zebrafish larvae [91]. This finding paves the way for theranostics of dopamine related diseases. However, a recent report elaborated on the toxicity of the gold nanoparticle as compared to ionic gold in zebrafish that were subjected to spiked sediment [64]. They reported that the nanoparticle altered neurotransmission in the zebrafish brain as it had an effect on the acetylcholine esterase activity. In an interesting work Sivaji et al. [198] aimed to deliver donepezil, a well-established drug for Alzheimer’s disease, through functionalized poly *N*-isopropyl acrylamide nanogels PNIPAM nanogel to the brain. They reported the gel could overcome the BBB and also showed sustained drug release using zebrafish model. This study therefore brings to the fore development of neurospecific nanogel for targeted drug delivery to the brain. The same group further reported synthesis of colloidal gold nanoparticle functionalized with polysorbate 80 and polyethylene glycol, with capabilities to overcome the blood–brain barrier for therapeutic purposes [199]. In this study they synthesized and validated a biocompatible nanocarrier with abilities to cross the blood–brain barrier and efficiently deliver neurospecific drugs.

**Table 2:** Nanoparticle-mediated drug delivery studies for neurodegenerative diseases using zebrafish

NP used	Insight	References
PEGylated and non-targeted NPs	Modulation of surface PEG chain lengths and NP size impacts endocytosis across the BBB and brain bioavailability of the NPs. This holds immense scope for future research in treating neurodegenerative diseases	[90]
SiNP	Evaluation of the neurotoxic effects of SiNP reveals its potential to cause PD	[211]
SiO_2_	Silicon oxide nanoparticles cause depression and anxiety and were used to create Parkinson’s like condition in adult zebrafish	[212, 213]
BmE-PtNPs	*Bacopa monnieri* Phytochemicals-mediated synthesis of platinum nanoparticles has neuroprotective effects in MPTP model of Parkinson’s disease	[214]
NP based strategy	The study evaluates the BBB penetration ability and bioavailability of several upconversion NPs in cell culture and zebrafish models	[215]
TiO_2_	Exposure to TiO_2_ NPs induces neurotoxicity and Parkinsonism in zebrafish larvae and PC12 cells	[216]
Au	Gold nanoparticle coupled with chaperone inhibits amyloid fibrils and ameliorates cognition in Zebrafish	[217]

A list of studies on neurodegenerative diseases using nanoparticles in zebrafish

## Translational Approach of Neurospecific Nanoparticles: Zebrafish to Humans

A variety of model organisms have been employed till date to investigate human diseases. While chimpanzees and monkeys have a high degree of similarity with humans, mice and rats have been used extensively over the past few decades. Research using zebrafish models to study various human diseases is now on the increase [31]. Various state-of-the-art technologies have been analysed and evaluated using the zebrafish model. In this context, nanodiamonds (ND) which refer to a newer class of nanoparticles belonging to the carbon family are being explored in the latest techniques for drug delivery across the BBB [200, 201]. They possess outstanding optical properties, malleability of surface structures and mechanical properties which are pertinent for targeted drug delivery. The zebrafish has proved to be an apt model system to study the fluorescent nanodiamonds (FND) in detail. Chang et al. have studied the photostability and non-toxicity of FNDs by single particle tracking using zebrafish yolk cells [202]. Further, evaluation of ND to facilitate their application as nanolabels has been performed using laser confocal microscopy and real-time fluorescence tagging in zebrafish [203]. Zebrafish model can hence be explored to assess the potential of NDs as nanolabelling systems to deliver neurospecific drugs. The use of zebrafish is validated by its high genetic and systems similarity with that of humans. Regenerative ability of zebrafish is also a very interesting aspect of its physiology which has made it an important model organism to study neurodegenerative diseases. Recent studies have identified pivotal insights into brain drug delivery mechanism using zebrafish models of neurodegenerative diseases. Recent research conducted regarding drug delivery in the brain using the zebrafish model has revealed pivotal insights about the dynamics of this mechanism. The only drawback withholding accelerated research in this arena is the lack of established protocols to validate the studies. However, it is only a matter of time when such protocols are developed through ongoing research in this field. A great deal of scope still exists for further research on the following focus areas.

- Admixture of nanoparticles along with two or more drugs to provide better holistic treatment
- Analysis of fullerenes, nano-onions and nanodiamonds in neurodegenerative diseases
- Understanding the biocompatibility of the newer nanoparticles and their brain-penetrating ability.

**Fig. 2:**
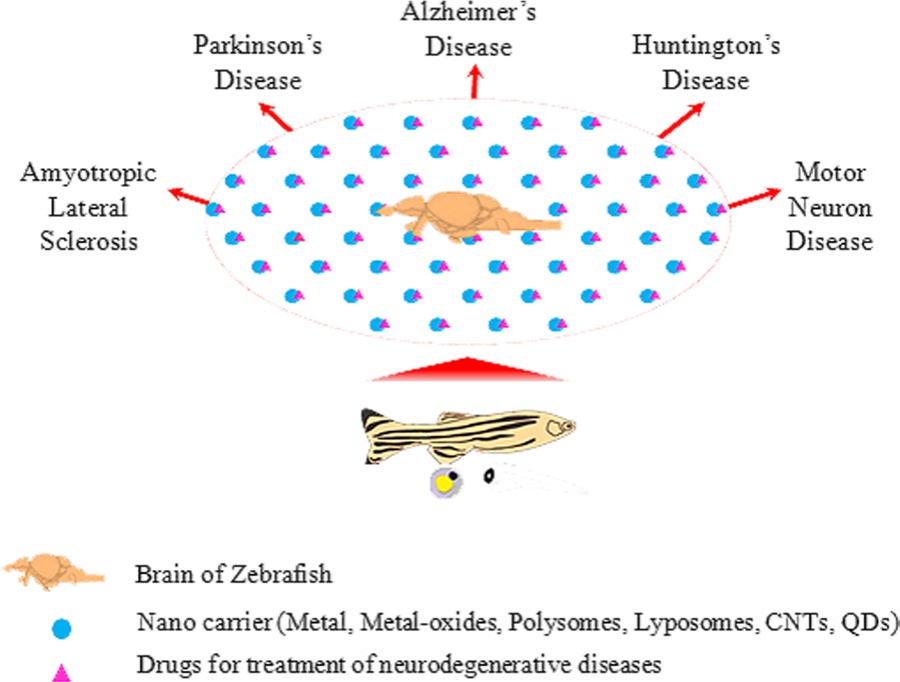
Schematic representation of zebrafish model for delivering drugs encapsulated in nanoparticles to the brain. This method ensures efficient delivery of drugs across the blood–brain barrier (BBB). Several nanoparticles possess the potential to treat a variety of neurodegenerative diseases like Alzheimer’s disease (AD), Parkinson’s disease (PD), Huntington’s disease (HD), amyotrophic lateral sclerosis (ALS) and motor neuron diseases (MND)

All the above-mentioned focus areas can be easily assessed using zebrafish model systems. The zebrafish model, therefore, holds great promise for development and evaluation of novel techniques for targeted drug delivery within the brain for translational analysis (Fig. 2). This could open up exciting new vistas for medical intervention to develop therapeutic strategies to treat neurodegenerative diseases.

## Future Research Directions

The last decade witnessed a surge in the use of nanotechnology for brain drug delivery unfolding several exciting new strategies in this arena [16, 17, 204, 205]. However, problems like toxicity, immunogenicity and efficient drug delivery still persist and have restrained the research community from achieving their ultimate goal [206–209]. Future research prospects for neurospecific drug delivery therefore involve overcoming the existing challenges in this field. Research on nanomaterial toxicity and side effects should be extensive, accurate and always preceed the in vivo implementation of any new nanocarrier formulation. Proper comprehensive analysis of the nano-bio-interactions is absolutely essential for developing strategies for neurospecific drug delivery [210]. Newer imaging techniques should be adopted to broaden the understanding of bio distribution and pharmacokinetics of the delivered drug. Complete knowledge on the bio availability and clearance of the drug is indispensable for achieving the translation from bench side to bed side. Zebrafish, long considered as a “gold standard” for studying several developmental and metabolic diseases, is highly prospective for studies on nanodrug delivery. The transparent embryonic development with the ability to facilitate large-scale drug screening in a vertebrate model among other innumerable key attributes of the zebrafish holds promise for overcoming these roadblocks. The use of this robust model system therefore has immense potential for further research in nanotherapeutics to achieve safe and successful neurospecific drug delivery.

## Conclusion

The BBB poses as the main obstacle in delivering drugs to the brain. The physiological function of the BBB is to protect the brain from foreign substances and in doing so it acts as a hurdle even for therapeutic purpose. The current need of the hour is a strategy in drug delivery which is able to overcome the BBB. Only then can effective treatments for brain specific diseases be possible. Recent focus on nanotechnology-based approaches for drug delivery across the BBB seems to have promising prospects for the field of neurospecific drug delivery in the future. Research towards this end is ongoing using a variety of nanoparticles like liposomes, dendrimers, micelles and carbon nanotubes as nanocarriers and nanogels. The zebrafish model is a favourite when it comes to nanotechnology-based toxicity studies and neurospecific drug delivery. Further research on nanotechnology using this model is needed for newer insights which can lead to possible breakthroughs in discovery in neurospecific drug delivery.

## Data Availability

Not applicable.

## Abbreviations

BBB: Blood–brain barrier
NPs: Nanoparticles
Au: Gold
Ag: Silver
Cu: Copper
Cd: Cadmium
CuO: Copper oxide
MgO: Magnesium oxide
NiO: Nickel oxide
ZnO: Zinc oxide
MPs: Microplastics
MOFs: Metal organic frameworks
CNTs: Carbon nanotubes
TiO_2_: Titanium dioxide
QDs: Quantum dots
PCR: Polymerase chain reaction
GQDs: Graphene quantum dots
PNIPAM: Poly *N*-isopropyl acrylamide
NDs: Nanodiamonds
FND: Fluorescent nanodiamonds
AD: Disease
PD: Parkinson’s disease
HD: Huntington’s disease
ALS: Amyotrophic lateral sclerosis
MND: Motor neuron diseases
